# Chemotherapy in elderly patients with early breast cancer: a systematic review

**DOI:** 10.1007/s00404-025-08120-5

**Published:** 2025-08-26

**Authors:** Miriam Fernández-Pacheco, Michael Gerken, Atanas Ignatov, Stephan Seitz, C. Kowalski, Elisabeth C. Sturm-Inwald, Maria Eleni Hatzipanagiotou, Olaf Ortmann

**Affiliations:** 1 Department of Gynecology and Obstetrics, University Medical Center Regensburg, Regensburg, Germany; 2 Comprehensive Cancer Center Alliance WERA (CCC WERA), Regensburg, Germany; 3 National Center of Tumor Diseases (NCT), Regensburg, Germany; 4 Tumor Center, Institute for Quality Management and Health Services Research, University of Regensburg, Regensburg, Germany; 5 German Cancer Society, Berlin, Germany; 6 Department of Gynecology and Obstetrics, Otto-Von-Guericke University, Magdeburg, Germany

**Keywords:** Elderly patients, Adjuvant chemotherapy, Neoadjuvant chemotherapy, Breast cancer

## Abstract

**Purpose:**

Breast cancer is the most common cancer affecting elderly patients. However, they may not receive optimal oncological care. Reasons might be comorbidities, limited compliance with clinical guidelines, and insufficient evidence for guideline recommendations. In the present review, the evidence for the clinical benefit of chemotherapy (CHT) in the population of elderly women with breast cancer was examined.

**Methods:**

A systematic review of relevant literature in English identifying studies published from January 2000 to April 2023 was conducted. The analysis included studies on the application of CHT, effects on survival, and toxicities. We searched PubMed databases for relevant publications. In total, 24 studies were included in the present review.

**Results:**

The benefit of CHT in elderly patients was inconsistent. Results of this review indicate evidence for the benefit of CHT in healthy elderly patients with high-risk breast cancer (BC) and specific subtypes such as triple-negative or HER2-positive BC. Data from studies on pathological complete response rates (pCR) or surgical downstaging rates after neoadjuvant chemotherapy (NACT) in different age groups are controversial.

**Conclusion:**

Results from studies on the effects of CHT in elderly patients are insufficient to draw differentiated conclusions due to heterogeneity of the definition for “elderly” patients, the application of different drugs and dosages. Patients with high-risk BC may benefit from CHT.

## Introduction

Breast cancer (BC) is the most common cancer affecting women older than 65 years. Demographic changes will lead to a larger population of elderly individuals with cancer [1]. Average life expectancy is currently almost 16 years for a 70- year-old woman and almost 7 years for a 85-year-old woman [2]. From 2010 to 2030, the percentage of cancer diagnosed in older adults will increase from 61 to 70% [3]. In addition, cancer patients are living longer. Therefore, the proportion of cancer survivors aged over 65 years will significantly increase over the next years [4]. In 2020, the 5-year overall survival (OS) rate was 88% for women with BC. Since the end of the 1990s, BC mortality rates have been falling steadily, especially among women between 60 and 69 years [5]. Older adults might be particularly vulnerable to suboptimal oncological care due to comorbidities, lack of adherence to clinical guidelines, and poor evidence due to underrepresentation of this subgroup in clinical trials [1]. This underlines the importance of research on cancer and aging. Regarding cancer treatment, especially CHT elderly patients often have a rather skeptical attitude. Some of the main reasons might be the underestimated life expectancy, the belief in a more favorable biologic behavior of cancer in elderly, a less favorable response to CHT, and fear of toxicity [6]. However, there is a significant lack of information on the safety and efficacy of cancer treatment for the growing numbers of older patients with cancer [7]. This becomes even more important since the biology of certain cancers may change with aging. Therefore, specific studies of the efficacy of treatment in dependence of age are necessary.

Regarding the application of CHT in elderly patients with BC, there are three main factors that have to be taken into consideration: (a) the effect of CHT on disease-free and overall survival, (b) the effect of different CHT regimens, and (c) toxicity and effects of treatment on quality of life. In this review, we analyzed the most relevant evidence for these questions.

## Materials and methods

A systematic search of literature was performed using PubMed databases identifying studies published in English language from January 2000 to April 2023. Studies were eligible for inclusion if they evaluated whether and to what extent elderly patients were treated with adjuvant or NACT. The following key words were used: “elderly patients”, “adjuvant chemotherapy”, “neoadjuvant chemotherapy”, or “breast cancer”.

For this review, we included different types of comparative studies, including randomized-controlled trials (RCTs), propensity score-matched analyses, retrospective studies, reviews, and meta‐analyses. Different definitions of “elderly” (older than 55, 65, 70 or 75 years) were accepted. BC stages ranged from 0 to III. Different CHT regimens according to current guideline recommendations were applied. After entering the keywords described above, 198 publications were identified. A selection process based on STROBE criteria was performed. Irrelevant records which did not deal with the topics “age” and/or “chemotherapy” were excluded, as well as studies that did not specifically analyze elderly patients or included only small cohorts of elderly patients. We found five studies analyzing the application of neoadjuvant CHT (NACT). The remaining trials analyzed the application of adjuvant CHT (ACT) in elderly patients or did not specify whether the therapy was adjuvant or neoadjuvant. Twenty-four publications analyzed the effect of CHT in elderly patients and were assessed for eligibility. Three systematic reviews, three meta‐analyses, six randomized-controlled trials (RCTs), two propensity-matched analysis, and twelve retrospective studies were included. The 24 selected studies were published between 2000 and 2023 with cohorts from 302 to 1,201,252 patients. Patients with breast cancer (BC) stages I–III and an indication for CHT were included in all studies. The distribution of the applied regimens of CHT differed between studies as well as the definition of “elderly (Table 1, see below). The following information was extracted from the studies (Table 1): author, year of publication, study design, publishing journal, sample size (*n*), outcomes of interest, neoadjuvant and adjuvant CHT, weaknesses of the trials, regimens of CHT applied.

**Table 1:** S tudies analyzing the application of neoadjuvant and adjuvant chemotherapy in elderly patients

StudyA1:M5L30A1:L6A1:M7A1:M8LA1:M22	Study type	Year of public	Journal	Cohort (n. of pats.)	Results	Neoadj./adj. chemotherapy	Weakness	Regimen of chemoth	Age groups
Terman et al.	Retrospective	2003	Breast Cancer Research and Treatment	2196 pats	Patients divided into groups based on race and age at diagnosis: Black women ≤ 40 y., White women ≤ 40 y., Black women ≥ 55 y., and White women ≥ 55 y. Young Black women had the highest risk of recurrence, 22% higher than young white women, 76% higher than older black women (*P* = 0.008). These age/racial differences in recurrence rates were not statistically significant after adjusting for subtype, stage, and grade. In terms of OS, older black women had the worst outcome. In the 397 women receiving NACT, 47.5% of young white women achieved pCR, compared to 26.8% of young Black women	Neoadj.	Unicentric, retrospective, small size of cohorts, no histological subtypes, no elderly	No regimens analyzed Comorbidity measured	2 groups: < 40 y and > 55 y
Giordano et al.	Retrospective study	2005	Journal of Clinical Oncol	1568 pats. With > 55 y	>75 y. less likely to receive treatment in concordance with guidelines for definitive surgical therapy, adj. radiation, adj. chemoth., only 62 pats 65-74 y and 22 pats >75 y received chemoth., respectively, 29,7 and 4% Overall concordance was lower for adjuvant chemotherapy (70.7%) and for adjuvant postmastectomy radiation (54.1%)	Adj.	Unicentric, retrospective, no possible survival benefit from chemotherapy analyzed, no different regimens/dosage etc.	No regimens analyzed, comorbidity index used	3 groups: 55 to 64 y. 65 to 74 y., and > 75 y
Elkin et al.	Retrospective cohort study, SEER data	2006	Journal of clinical Oncology	5,081 pats. with chemoth. with HR-negative BC >66 y	Relationship between adj. chemoth. and survival in a large, total cohort of 1,711 (34%) of 5,081 pats. with chemoth. use decreased with age and comorbidity, and increased with year of diagnosis, tumor size, n. of pos. lymph nodes, and higher tumor grade. adj. chemoth. associated with 15% mortality reduction, greatest OS benefit observed in patients with N+ disease.	Adj.	No different regimens/dosages etc., tolerability by age group (</>70y) not analyzed	No regimens analyzed Comorbidity measured	5 groups: 66-69 y., 70-74 y., 75-79 y., 80-84 y., >85 y.
Kaplan et al.	Retrospective cohort study	2016	The Breast Journal	771 pats. with TNBC	80% <65 y (n = 612), 13% were 65-74 y. (n = 100), and 7% > 75y (n = 59). Older women lower stage BC, less often treated with adj. or neoadj. (10%) chemoth. and less likely to be treated with a AC-containing regimen. Five-year DSS was equivalent across the three age groups, age was not significantly associated with DSS despite less aggressive treatment in patients 75 y. and older	Adj.	Unicentric, cohort, cohort of > 75y very small, no specification of regimens/no dosages analyzed	AC- and taxane-containing regimens No comorbidity index measured	3 groups: < 65 y., 65-74 y., > 75y.
Liedtke et al.	Retrospective cohort study	2013	Breast Cancer Res Treat	1732 pats. with TNBC	Comparison between age cohorts (max. >60y). Effect of age on DFS, DDFS and OS. Increasing age less tumor grade, less chemoth. and longer/better DFS, DDFS and OS. Clinical characteristics of TNBC differ by age group, patients <40 y. have poorer survival despite more aggressive therapy. pCR similar across age groups	Adj. and neoadj.	Unicentric, “elderly” > 60y, no more differentiation, no survival analysis with consideration of regimens	AC/AC–taxane, CMF, taxane No comorbidity	5 groups (<30 y., 31–40 y., 41–50 y., 51–60 y., and >60 y
Kozak et al.	Retrospective	2018	The Breast Journal	19632 pats. with TNBC, data of SEER	>70 (n = 4221) less likely to receive chemoth. and higher BCSM compared to younger, stage II patients derived the greatest relative and absolute benefit from adj. treatment. Only 1780 (42.2%) >70, received chemoth. compared to 12811 (83.1%) younger, elderly benefit from more aggressive therapy of TNBC, and undertreatment, rather than age, is in part responsible for worse outcomes	Adj	Retrospective, reasons for undertreatment of the elderly were not evaluated, no recurrence rates, no regimens of chth.	No regimens analyzed No comorbidity index measured	2 groups: 18‐69 y. and > 70 y.
Syed et al.	Retrospective study	2014	PLoS ONE	1,758 pats. with TNBC (biological characteristics of tumors and survival analysis)	127 > 70 y. lower rates of Ki67 and CK 7/8 positivity compared with their younger counterparts, no significant difference in the long-term clinical outcome between the two age groups, despite the fact that 47% of the younger patients had adj. chemoth. and none in the older cohort. >70 more low-grade tumors, reduction of Ki67 expression, more frequent normal p53 and higher expression of Bcl2.	Adj.	Unicentric, retrospective	No regimens analyzed No comorbidity measured	2 groups: <70 y. and > 70 y.
Pierga et al.	Retrospective study	2004	The Breast	1755 > 70 y. pats.	Adj. endocrine therapy given in 463 (26%) cases, and only 3% of patients received chemoth. Median OS time was 121 months. The overall cancer-related death rate was 49%. The 10-year disease-free survival (DFS) rate was 64%, and the 10-year local relapse rate was 14%. Prognostic factors were tumor size, nodal status, ER/PR, and grading. BC in elderly is frequently HR-pos. (81%) with a low proliferation index. Prognostic factors are the same as in younger postmenopausal patients. More than 50% of these patients died from a cause other than their BC.	Adj.	Retrospective, only 3% received chemotherapy, no comparison/analysis on the effect of chemoth. on survival	No regimens analyzed No comorbidity measured	5 groups: 71–75 y., 76–80 y., 81–85 y., 86–90 y., 91–94 y.
Hurria et al.	Retrospective study	2003	Critical reviews in Oncology /Hematol ogy	216 patients age > 75 y	46 pats. were 75-79, 50 were > 80, two independent prognostic variables for not receiving combined local treatment: increased age and increased comorbidity score. Increased age did not correlate with increased comorbidity. 5.2% of pats. received adj. chemoth. (all age > 80). Both comorbidity and age play a significant role in influencing treatment decisions in the elderly, but these two variables are not necessarily correlated.	Adj.	Unicentric, only 5,2% received chemoth., small cohort	No regimens analyzed Comorbidity measured	2 groups: 75-79 y., >80 y.
Woodard et al.	Retrospective study	2005	Journal of Clinical Oncology	480 pats., National Comprehensive Cancer Network	<50 y (n = 143 [30%]), 50-65 y (n = 216 [45%]), and > 65 y. (n = 121 [25%]). Odds of not receiving chemoth. for 50-65 y and >65 y with ER+BC approximately 6 times greater than the odds for <50. 50-65 y with ER-negative BC not significantly different from < 50 y. with respect to chemotherapy use, >65 y. with ER-negative BC 7 times less chemoth. than <50 y. elderly less likely to receive adj. chemoth. Charlson Comorbidity Index and baseline performance status not identified as confounders in decisions regarding adj. chemth.	Adj.	only 21 pats > 65 y received chemoth. (17%), no regimens analyzed, no survival benefit measured, small cohort, retrospective	No regimens analyzed, comorbidity measured	3 groups: <50 y., 50-65 y., > 65 y.
Inwald et al.	Retrospective	2017	Breast Cancer Res Treat	3463 patients, multicentric	Comparison: 50–69 y (2171 pats.) and 1292 patients >70, subtypes comparable in both groups. Less systemic therapies in all subtypes in > 70. In Luminal A, best OS in pats. receiving CHT+ET <70. In >70, best OS was seen in CHT+ET (95.2%), whereas pat. receiving only ET had a OS of 73.9%. Despite similar tumor biology, elderly patients are undertreated regarding systemic and local therapies compared to younger patients, leading to reduced OS	Adj.	Only 187 women >70 received chemoth	No regimens	2 groups: 50–69 y., >70 y
Inal et al.	Retrospective	2013	Journal of Balkan Union of Oncology	191 patients >70y	Efficacy chemoth. with ET vs. ET alone in ER+ and N+ BC, 130 pats. received chemoth., 114 pats. were 70-80 y old, 16 pats > 80 y. 30-month DFS rates were 50.0% in the ET arm and 49.0% in the chemoth/ET arm (*P* = 0.79). The 30-month OS rates were 86% in the ET arm and 96.0% in the chemoth/ET arm (*P* = 0.08). Cox proportional hazard model showed that only surgery was an independent prognostic factor for survival (*P* = 0.047); in addition of chemoth. to ET in older patients, no significant impact on DFS and OS	Adj.	Retrospective, unicentric, follow-up time only 29 months, small cohorts, molecular characteristics of tumor not evaluated, no dosage taken into account, heterogenous regimens (no comparison)	4 diff. regimens (+/- taxane) Comorbidities taken into account	2 groups: 70-80 y., >80 y.
Williams et al.	Retrospective	2022	Ann Surg Oncol	651	≥70 ↓AC-based NAC, ↓completion of regimen, high rates of breast/axillary downstaging (pCR), similar between age groups	Neoadj.	Very small cohort of >70, selection bias (healthy elderly receive. NAC), NAC regimens were not standardized	AC-T, TC, CMF or TzPz and no HER2-specific therapy Comorbidity score used	2 groups: 50-69 y., ≥70 y.
Plichta et al.	Retrospective	2020	Breast Cancer Res Treat.	1,201,252 total cohort (< 45 and > 75 compared) N=210,095 >75 y	c/p T/N stages significantly more advanced in younger. Rates of de novo cM1 comparable. Younger were more likely to receive chemoth (65.8% vs. 10.2%, *P* < 0.001). ≥ 75 y. worse unadjusted and adjusted OS in comp.	No diff.	No adjustment for therapy/therapy regimen, no treatment specific mortality	Not adjusted for regimens Comorbidity score used	3 groups: 18-45 y., 46-74 y. ≥ 75 y.
Qiusheng et al.	Retrospective SEER database	2023	Biomol Biomed	4610 elderly patients (70-90y)	Elderly pats. with TNBC benefit from adj. chemoth., BCSS and OS in the chemoth. (n = 1989) group higher (*P* = 0.010, *P* < 0.001) in comparison with the observation (n = 2621)) group, percentage of patients receiving AC vs. observation increased significantly by time, pats. with T1a could not benefit from AC (*P* > 0.050)	Adj.	Regimen changes during 2010–2018 not taken into account, lack of detailed chemotherapy regimens information, no comorbidity index/toxicities	Not adjusted for regimens Comorbidity score not used	2 groups: 18–69 y., > 70 y.
Janeva et al.	Propensity score-matched analysis	2020	Lancet Health y Longev	1130 pats. > 70 y. SNBCR among others	368 (32.6%) received adj. chemoth., 45 (4,0%) neoadj. th. and 717 (63,5%) no chemoth. BCSS significantly improved in pats. who received adj. chemoth. (85% [95% CI 81–89] vs. 68% [64–72]; (*P* < 0ꞏ0001), treatment with adj. chth. significant benefit in terms of BCSS and OS, also when adjusting for age and comorbidities	Adj. and neoadj.	Number of pats. with neoadj. chemoth. too small, no different regimens or dosages of chemoth. analyzed	Not adjusted for regimens	3 groups: 70-74 y., 75-79 y., > 80 y.
Fargeot et al.	Prospective	2004	J of Clinical Oncol	338 patients > 65 y.	In > 65 y with axillary lymph node involvement epirubicin (EPI)-based chemoth +hormonal regimen improves DFS relative to tamoxifen (TAM) alone. 6-year DFS rates were 69.3% with TAM and 72.6% with EPI-TAM. 6-year OS related to disease progression was 79.1% and 79.8%. Compliance with chemoth. was good with 96.9%, acute toxicity was mild (grade 2 neutropenia or anemia, grade 3 nausea or vomiting and grade 3 alopecia). Five cases of decreased left ventricular ejection fraction occurred after chemoth. significant contribution of a weekly EPI regimen in terms of DFS, safe for hematologic, non-hematologic, and cardiac toxicities. At a median follow-up of 72 months, an OS advantage with the combination regimen was not observed. No difference in OS between age groups	Adj.	No analysis of histological type, “elderly women” age > 65 y., selection bias (WHO performance status > 2, normal hematologic, hepatic, and renal functions, and no cardiac dysfunction), small cohort (only 174 EPITAM), only 71 pats > 70 y. received chemo	Epirubicin-only regimen analyzed Comorbidity measured	2 groups: <70 y., > 70 y.
EBCTCG (effects of chemotherapy and hormonal therapy)	Meta-analysis of 194 RTs (six meta-analyses)	2005	Lancet	36000 pats receiving chemth., 194 RTs 6 meta-analyses: AC-based vs. no chemoth. (*n* = 8000); CMF-based vs. no chemtoh. (*n* = 14000), AC-based vs. CMF-based (*n* = 14000)	6 months of AC-based polychemoth. Reduce annual BC death rate by about 38% in pats.< 50 and by about 20% in 50–69 y, irrespective of the use of tamoxifen and of ER status or nodal status. Such regimens are significantly more effective than CMF. BC mortality rate halved by 6 months anthracycline-based chemotherapy followed by 5 y. of adjuvant tamoxifen. 17/996 and 8/1143 (polychemo vs. adjusted control in > 70 y), non-significant excess of deaths during the first 2 y. among women of age 60–69 y. or 70 and older, suggesting early hazards of 0ꞏ2% and 2%, respectively. These trials of chemotherapy involved too few women older than 70 y. of age to be reliably informative as to whether it confers any survival benefit	Adj.	No regimen containing taxanes, trastuzumab or modern aromatase inhibitors, few pats. > 70 y entered these chemoth. trials	Regimens: CMF, anthracycline-based combinations such as FAC or FEC, tamoxifen or ovarian suppress ion No comorbidity index measured	4 groups: < 50 y., 50–69 y., 60–69 y., > 70 y.
EBCTCG (comparison polychemoth.)	Meta-analysis of RTs	2012	Lancet	101.000 patients	Addition of taxan is a benefit even at 55–69 y, few pats. were older, but their results suggest favorable effects of taxanes even in old age, but with wide uncertainty. Involving taxane-based regimens and AC-based regimens reductions in recurrence and BC mortality shown independently of age, stage, grading ,and tumor biology. Few pats. > 70 y. entered these trials; they may have somewhat greater immediate hazards from chemoth., but as great a reduction as younger in BC recurrence and mortality.	Adj.	Few pats. >70, no comorbidities taken into account	Taxane+ AC-based regimen, CMF, CAF, CEF	4 groups: <50 y., 50–69 y., 60–69 y., > 70 y.
EBCTCG (long-term outcomes NACT vs. ACT)	Meta-analysis from 10 RT	2018	Lancet Oncol	4756 women with BC	Comparison NAC with the same adj. chemoth., >2/3 (1349 [69%] of 1947) of women with NACT had a complete/partial clinical response. 15-year local recurrence was 21ꞏ4% for NACT vs. 15ꞏ9% for adj. chemoth. (5ꞏ5% increase [95% CI 2ꞏ4–8ꞏ6]; no significant difference between NACT and adj. chemoth. for distant recurrence (15-year risk 38ꞏ2% for NACT vs. 38ꞏ0% for adjuvant chemotherapy) or for BC mortality (34ꞏ4% vs. 33ꞏ7%; 1ꞏ06 [0ꞏ95–1ꞏ18]; *P* = 0ꞏ31), or death from any cause (40.9% vs. 41.2%; 1.04 [0.94–1.15]; *P* = 0.45). Tumors downsized by NACT might have higher local recurrence after BCT. Age, nodal status, and planned local therapy did not affect response.	NACT and adj.	> 55 “oldest group”, no proper analysis of ,”elderly“	Most chemoth. AC-based (81%), no comorbidities analyzed	3 groups: < 45 y., 45–54 y., > 55 y.
Balducci et al.	Review	2001	Cancer Control	24 trials (chemoth. In postmenopausal women)	Adj. chemoth. beneficial to pats. with HR-poor tumors. In those with HR-rich tumors, adj. chemoth. is beneficial for HER2+ tumors, and the regimen should contain an AC. All > 70 should undergo geriatric assessment, drugs whose compounds/metabolites excreted from the kidneys should have the dose adjusted , prophylactic use of hemopoietic GF recommended for > 70, Hb should be >12 g/dL, anemia associated with functional dependence and cardio-cerebrovascular complications, in >70 y, adj. chemoth. beneficial for survival if risk of relapse is >10% and for >80y, when > 21%. On the other hand, influence of age on the benefit in relapse is low.	Adj.	Heterogenous data on regimens, dosages, with/without comorbidity index, “elderly” defined as > 50 y in some trials, older regimens (not currently administered)	Heterogenous regimens analyzed, some trials used comorbidity scores	Not clearly defined
Ring et al.	Minireview	2011	British Journal of Cancer	4 randomized-controlled trails CALGB 49907, CALGB 9741, EBCTCG, ICE	EBCTCG: benefits of polychemoth. Decrease with increasing age but the benefit for > 70 y was of the same order as for younger (CAVE result not significant as the cohort of elderly was small) CALGB 49907: comparison CMF/AC to oral capecitabine. 61% of pats. > 70 y. After 2 y, pats. with capecitabine 2.4 times more recurrence and 2.1 times more deaths than those receiving standard chemoth. CALGB 9741 trial: benefits of dose- dens, only 3% of pats > 70. Taking into account, higher toxicity rate, review of 4 CALGB trials found treatment-related mortality related to age: 0.2% (< 50 y.), 0.7% (51–64 y.)and 1.5% (> 65 y.).	Adj.	Older study, smaller cohorts of > 70 (“elderly”) often defined as > 65), old regimens (not used any more)	Different regimens comorbidities only partially taken into account	Different trials analyzing patients > 65 y and >70 y. Old
Muss et al.	RCT	2007	J Clin Oncol	6642	Comparison of toxicity of ACT in elderly and younger patients with node-positive BC including data from three trials: CALGB 8541, CALGB 9344 and CALGB 9741. Elderly patients significantly more likely to have gr. 4 hematologic toxicity, more treatment-related deaths, discontinue treatment for toxicity or to die of AML. No significant differences in non-hematologic toxicity Elderly patients with ACT seem to have same benefits as younger patients but increased risk of toxicity and treatment-related death	ACT	Only 7% of patients were “elderly” (> 65 y.) and only 3% were > 70 y	3 different regimens: cyclophosphamide (C), doxorubicin (D), and fluorouracil (F) in three dose schedules; C+D +/- paclitaxel (P); C+D+P every 2 vs. 3 weeks	3 groups: 65-69 y., 70-79 y., > 80 y
Crivellari et al.	Randomized trial (International Breast Cancer Study Group Trial VII)	2000	J Clin Ocol	608 patients with N+ BC	Pats. randomized to receive tamoxifen alone (306 patients) or tamoxifen + CMF (302 patients). > 65 y higher toxicity compared to younger. Older patients received less than their expected CMF dose compared with younger. Subjective burdens of treatment were similar for younger and older patients based on quality-of-life measures. For older patients, DFS rates were 63% for CMF + tamoxifen and 61% for tamoxifen alone. For younger patients, DFS rates were 61% and 53%, but the test for heterogeneity of CMF effect according to age group was not statistically significant. The reduced effectiveness of CMF among older could not be attributed to dose reductions. CMF tolerability and effectiveness reduced for older compared to younger	Adj.	Small cohort of “elderly” (only 119 pats from 65-69y), only 53 pats > 70 y.	Regimen: tam. vs. CMF no comorbidities taken into account	2 groups: <65 y., > 65 y.
Citron et al.	Randomized trial	2003	Journal of Clinical Oncology	2005 pats.randomized with nodal positive BC	Comparison of sequential doxorubicin, paclitaxel, and cyclophosphamide with concurrent doxorubicin and cyclophosphamide followed by paclitaxel for DFS and OS and toxicities. Dose-dense treatment improved DFS (risk ratio [RR] 0.74; P .010), and OS (RR 0.69; P .013). Four-year DFS was 82% for the dose-dense regimens and 75% for the others. There was no difference in DFS or OS between the concurrent and sequential schedules. Dose density improves clinical outcomes significantly, sequential chemotherapy is as effective as concurrent chemoth.	Adj.	Small cohorts, only 298 pats. 60-69 y (max. 17%), 50 pats. > 70 (max 3%), no stratified analysis after age	Regimens: doxorubicin, paclitaxel and cyclophospham. No comorbidities taken into account	2 groups: 60-69 y., > 70 y
Albain et al.	RT phase III trial	2009	Lancet	1477 postmenopausal women with HR-pos., N+ BC	CAF superior to T for DFS (*P* = 0.002), CAF followed by T marginally better than CAF with simultan. T for DFS (*P* = 0.055). Ten-year DFS for CAF-T, CAFT, and T were 60%, 53%, and 48%, respectively. CAF better for OS in comparison to T [*P* = 0.043, adjusted HR 0.83 (0.68,1.01)] Chemoth. with CAF+T longer survival over T in HR+, N+ BC	Adj.	Small cohort of pats: 190 pats (13%) > 70, only 11% and 15% represented in the chemoth- groups, old regimen (not used any more)	Regimens: CAF vs. Tamoxifen alone No comorbidities analyzed	2 groups: 65-70 y., > 70 y
Perrone et al.	Multicenter, randomized phase III study	2015	Ann Oncol	302 patients	Comparison weekly docetaxel vs. standard chemoth. in 65–79 y in terms of DFS. After 70 months, 109 DFS events. HR of DFS for docetaxel vs. CMF 1.21, *P* = 0.32; DFS estimate at 5 y. 0.69 with CMF and 0.65 with docetaxel Hematological toxicity, mucositis, and nausea worse with CMF; allergy, fatigue, hair loss, onychopathy, dysgeusia, diarrhea, abdominal pain, neuropathy, cardiac and skin toxicity worse with docetaxel. 1 death attributed to CMF and 2 to docetaxel. Increasing age, impairment in instrumental daily living activities, n. of comorbidities and docetaxel treatment associated + severe non-hematological toxicity. QoL worse + docetaxel, weekly docetaxel not more effective than CMF as adj.treatment of elderly and worsens QoL and toxicity	Adj.	Quality of life (QoL) was assessed, cohorts small	Regimens: docetaxel vs. CMF Comorbidities measured, geriatric assessment carried out	3 groups: 65-69 y., 70-74 y., 75-79 y.
Pagani et al.	Randomized trial, International Breast Cancer Study Group (IBCSG)	2009	Breast Cancer Res Treat	893 patients with endocrine-responsive disease	Comparison efficacy chemoendocrine treatment+ ET alone for postmenopausal. chemoth. Reduced RR by 19% (*P*=0.02) compared to ET alone, little effect of chemoth. for tumors+ high ER expression (*P* = 0.07) and nodal positive BC (*P* = 0.03). Chemoth. significantly improves DFS for HR+ BC, but effect attenuated by high ER. RR reduced by 22% and 17% in < 60 y. and >60 y., and by 11% and 31% in pats.< 3 or > 4 positive nodes, benefit of chemoth. greater in < 60 y.	Adj.	”elderly“ >60 y, only 2 different regimens, no OS, no stratification in comorbidities, no toxicity analyzed	Regimens: CMF (mostly) and AC no comorbidities analyzed	2 groups: <60 y., > 60 y.
von Minckwitz et al.	Randomized Phase 2 Study	2015	Cancer.	391 pats. > 65	Pats. randomized for 4x EC or 6 x CMF vs. 6 x nab-paclitaxel and capecitabine (nPX). 13 of 198 patients (6.6%) discontinued EC/CMF and 69 of 193 patients (35.8%) discontinued nPX (*P* < 0.001) with 1 and 5 deaths during treatment, respectively. Grade 3 to 5 adverse events more frequent among pats. treated with EC/CMF (90.9%) than with nPX (64.8%) (*P* < 0.001), with hematological toxicities being more frequent with EC/CMF (88.4% vs. 22.3%; *P* < 0.001), but non-hematological toxicities more frequent with nPX (58.5% vs. 18.7%; *P* < 0.001). None of the geriatric scores independently predicted grade 3 to 5 toxic events or treatment discontinuations No differences in survival between the treatment groups were observed after 22.8 months compared with EC/CMF, treatment with nPX led to more treatment discontinuations and non-hematological toxicities in elderly	Adj.	Stopped after phase 2 part and did not proceed to phase 3 to adequately assess survival in both treatment arms	Comorbidity index measured	3 groups: 60-69 y., 70-80 y., > 80 y

## Results

### Effect of CHT in elderly patients

#### Triple negative breast cancer

The effect of CHT in elderly patients was analyzed in nine studies. Of these, eight studies only analyzed triple-negative BC (TNBC) in elderly patients, the other four studies mostly analyzed high-risk BC (e.g., positive axillary lymph nodes). Williams et al. compared rates of downstaging by CHT in patients regarding breast-conserving-surgery (BCS) eligibility and avoidance of axillary lymph node dissection (ALND) between patients aged 50–69 and ≥70 years [8]. On the one hand, women older than 70 years were less likely to have lobular cancers, received less adriamycin and cyclophosphamide** (**AC)-based NACT and were less likely to complete the prescribed regimen. On the other hand, they experienced high rates of breast and axillary downstaging and nodal pCR. This suggests that in well-selected elderly patients, NACT is safe and has a substantial clinical benefit, with pCR as a surrogate parameter for survival. The main weakness of this study was the small cohort of elderly patients, with only 75 (11%) patients older than 70 years. Williams et al adjusted for Charlson Comorbidity Index finding no difference between age groups.

Qiusheng et al. analyzed data from 4610 elderly patients (70–90 years) with TNBC and compared patients receiving ACT with those who did not [9]. Data was extracted from the Surveillance, Epidemiology and End Results (SEER) database and covered 18 regions—approximately 34.6% of the U.S. population. Breast cancer specific survival (BCSS) and overall survival (OS) in the ACT group (*n* = 1989) and observation group (*n* = 2621) were compared. The percentage of patients receiving ACT vs. observation increased significantly from 2010 to 2018 (estimated annual percentage change, 1.49%; 95%CI, 0.75–2.16%, *P* = 0.002). Inverse probability of treatment weighting (IPTW) was performed, a technique to adjust for confounding in observational studies using propensity scores. 5-year IPTW-adjusted rates of BCSS and OS and IPTW-adjusted Cox proportional hazards regression analysis were better in the ACT group than in the observation group (BCSS: HR 0.77, 95%CI 0.62–0.94, *P* = 0.012; OS: HR, 0.66, 95%CI, 0.57–0.78, *P* < 0.001). They found patients with lower tumor stages of T1a/b of any age would not benefit from AC (*P*> 0.050).

Crozier et al. performed a propensity-matched analysis with data from the National Cancer Database on 16062 women older than 70 years with operable TNBC [10]. In total, the general 5-year OS was 62.3% (95% CI 59.7–64.4). Regarding subgroups, the 5-year OS for patients receiving CHT was 68.5% (95% CI 66.4–70.6), 61.1% (59.0–63.2) for patients recommended but not given CHT, and 53.7% (51.8–55.8) for patients for whom CHT was not recommended or applied. Multivariable Cox regression analysis comparing patients who received CHT with those who did not (*n* = 1884) found improved OS with CHT (HR 0.69 [95% CI 0.60–0.80]; *P* < 0.0001). This beneficial effect was independent of nodal status and comorbidity score and supports the consideration of applying CHT in patients older than 70 years with TNBC.

Janeva et al. analyzed data from 1130 patients older than 70 years with primary early TNBC in a population-based registry study including the Swedish National Breast Cancer Registry, the Swedish Patient Registry, and the Swedish Cause of Death Registry [11]. A propensity score-matched model was used to examine outcomes after ACT such as 5-year BC specific survival (BCSS) and 5-year OS, adjusted for confounders like comorbidities. The outcomes of three groups were compared: 368 patients (32.6%) received ACT, 45 patients (4.0%) received NACT, and 717 patients (63.5%) did not receive CHT at all. In comparison with patients who did not receive CHT at all, 5-year BCSS was significantly improved in patients who received ACT (85% [95% CI 81–89] vs. no ACT 68% [64–72]; *P* < 0.0001). The same observation was made for 5-year OS in patients treated with ACT in comparison with those who were not (75% (95% CI 69–82) vs. 63% (57–71; *P* = 0.029)). This effect persisted even when adjusting for age and comorbidities. These results suggest the importance of considering ACT in older patients with TNBC. However, the cohort of patients who received NACT was too small to draw clear conclusions. In addition, there was no differentiated analysis for regimens and/or dosages of CHT.

Elkin et al. assessed the relationship between ACT use and survival in a large, population-based cohort of patients with BC older than 66 years with HR-negative, non-metastatic BC [12]. From a cohort of 5081 women with HR-negative BC, 1711 (34%) received ACT. Use of CHT decreased with increasing age and comorbidities and increased with year of diagnosis, tumor size, number of positive lymph nodes, and higher tumor grade. ACT was associated with a reduction of mortality by approximately 15% whether analyzed using propensity scores or standard multivariable methods. The greatest OS benefit was observed in patients with node-positive disease. This analysis suggests a survival benefit from ACT in older women with HR-negative BC. The benefit of CHT is stronger in patients who had positive lymph nodes or other high-risk disease characteristics.

Kaplan et al. explored the mortality risk of elderly patients with TNBC compared to younger women related to treatment [13]. Data from 771 patients with primary TNBC were analyzed. Of all patients, 80% were younger than 65 years (*n* = 612), 13% were between 65 and 74 years old (*n* = 100), and 7% were older than 75 years (*n* = 59). Older women presented more often with lower stages of BC (stage I: 31% patients older than 65 years, 48% older than 65–74 years, 39% older than 75 years, *P* = 0.014). All three age groups had similar rates of radiotherapy (77%). However, older patients were less often treated with ACT (95% were younger than 65 years, 76% were between 65 and 74 years and 39% were older than 75 years: *P *< 0.001). Mean of follow-up was 7.34 years. Five-year disease specific survival (DSS) was equivalent across the three age groups (85% in patients younger than 65 years, 90% in patients with the age of 65–74 years and 83% in patients older than 70 years, *P* = 0.322). In Cox regression analysis, age was not significantly associated with DSS. Contrary to the studies mentioned above, TNBC survival appears equivalent by age despite less aggressive treatment in elderly patients, although the cohort of elderly patients was small and no specific regimens were analyzed.

Liedtke explored the prognostic impact of age in 1732 patients with primary TNBC from the Breast Medical Oncology Clinical Database, 5 subgroups with different ages were analyzed (younger than 30, 31–40, 41–50, 51–60, and older than 60 years) as well as the effect of age on DFS, distant disease-free (DDFS), and OS [14]. They found that increasing age at diagnosis was inversely correlated with tumor grade (*P* = 0.0001), likelihood of getting CHT (*P* = 0.0001); and positively correlated with favorable DFS (*P* = 0.0003), DDFS (*P* = 0.0001), and OS (*P* = 0.0001). The DFS and OS were also significantly shorter for younger patients. In multivariable analysis, tumor size, nodal stage, tumor grade, and age remained significant independent prognostic variables. Clinical characteristics of TNBC differ by age group, with younger patients having poorer survival rates despite more aggressive systemic therapy. Patients who were diagnosed at age younger than 30 or 31–40 years received NACT (38 and 35 vs. 26 %) or ACT (65 and 61 vs. 43 %) significantly more frequently than patients older than 60 (*P* = 0.0012 and *P* = 0.0001, respectively). Among the patients who received ACT, patients younger than 30 years at diagnosis were significantly more likely to receive an AC-containing combination CHT compared to patients older than 60 years (44 vs. 29 %, *P* = 0.0001). Regarding the smaller group of patients receiving NACT, pCR rates after NACT did not significantly vary across different age groups (*P* = 0.87). In older patients, better OS and DFS rates were reported despite less aggressive systemic therapies, which questions the role of pCR as a surrogate parameter for OS.

Kozak et al. identified patients with TNBC using the SEER database [15]. Patients older than70 years (*n* = 4221) were less likely to receive CHT, radiation therapy (*P* < 0.0001) and had higher breast cancer specific mortality (BCSM) in general compared to younger women (12.8% at 3 years vs. 10.2% *P* < 0.0001) with a relative increase in mortality of 25%. There were no statistically significant differences in BCSM in the subgroup of patients who received ACT (*P* = 0.10), the subgroup of patients with BC stage II showing the greatest relative and absolute benefit from adjuvant treatment. Younger patients appeared to have the greatest relative and absolute benefit from adjuvant CHT regarding BCSM. One hypothesis for this result is that women older than 70 years may have received less aggressive CHT regimens and fewer CHT cycles compared to younger women. The regimens applied were not analyzed in this study.

Syed analyzed tumor characteristics and CHT effects in patients with early TNBC [16]. One hundred twenty-seven patients (22.1%) were older than 70 and three hundred forty-two (18.9%) younger than 70 years. Older patients showed lower rates of Ki67 and CK 7/8 positivity and high rates of bcl2 and CK18 positivity compared to younger patients (*P* = 0.05). There was no significant difference in long-term clinical outcome between the two age groups, despite the fact that 47% of the younger patients received ACT, while none of the patients in the older cohort received such treatment. There was even a trend of slightly better survival among elderly patients (5-years BCSS of 73% in younger patients vs. 79% in elderly patients). EGFR, axillary stage, and tumor size showed prognostic significance in older women with TNBCs in univariate analysis. Although older patients did not receive CHT, they had similar outcomes, which appears to be related to certain biomarkers (in addition to ER/PgR/HER2), e.g., Ki67, bcl2, and cytokeratins which have different expression patterns influencing prognosis. Older patients had more low-grade tumors, reduced expression of Ki67, more frequent normal p53, and higher expression of Bcl2.

#### Hormone receptor (HR)-positive, node-positive and HER2-positive breast cancer

Inwald et al. analyzed data from a population-based clinical cancer registry of 3463 BC patients [17]. The distribution of tumor biological subtypes was evaluated among patients aged 50–69 years and older than 70 years. Local and systemic therapies in different subtypes as well as OS were analyzed. 2171 patients were younger than 70 years of age, 1292 patients were older than 70 years of age. Distribution of BC subtypes was comparable in both groups. Treatment varied considerably with less systemic therapies in all subtypes in patients older than70 years. In patients with luminal A BC, best OS was seen in patients receiving endocrine therapy (ET) or CHT plus ET in patients younger than 70 years. In patients older than 70 years, best OS was seen in CHT plus ET (7-year OS of 95.2%), whereas patients receiving only ET had a 7-year OS of 73.9%. Also, in elderly patients with luminal B tumors, adjuvant therapy (endocrine treatment ± CHT) improved outcomes. The authors concluded that despite similar tumor biology, elderly patients are undertreated regarding systemic therapies compared to younger patients leading to reduced OS. Biology of tumors did not significantly differ among elderly and younger patients. In all subtypes, OS was lower in elderly patients compared to younger patients. However, the effect of chemotherapy in elderly patients lowering their cancer-related OS was similar as in younger patients.

Pagani et al. also compared the efficacy of chemoendocrine treatment with that of endocrine treatment alone for postmenopausal with highly endocrine-responsive BC [18]. In the International Breast Cancer Study Group (IBCSG) Trials VII and 12-93, 893 postmenopausal women with node-positive, estrogen receptor (ER)-positive or ER-negative, operable BC were randomized to receive either CHT or endocrine therapy or combined chemoendocrine treatment. Adding CHT reduced the relative risk of a disease-related event by 19% (*P* = 0.02) compared with ET alone. This significant improvement in DFS by CHT was attenuated showing little effect of CHT for tumors with high levels of ER expression (*P* = 0.07), or for the cohort with one positive node (*P* = 0.03).

Inal et al. analyzed 191 patients older than 70 years with hormone-receptor-positive and node-positive BC regarding the efficacy of adjuvant endocrine therapy (ET) vs. ACT with ET [19]. 30-month DFS rates were 50.0% in the ET arm and 49.0% in the CT/ET arm (*P* = 0.79). The 30-month OS rates were 86% in the ET arm and 96.0% in the CT/ET arm (*P* = 0.08). Cox proportional hazard model showed that only surgery was an independent prognostic factor for survival (*P* = 0.047), while tumor size showed a strong trend for statistical significance (*P* = 0.051). The addition of CT to ET in older patients showed no significant impact on DFS and OS in this study. However, neither applied dosages nor regimens were analyzed in this study.

### Effect of different CHT regimens

Albain et al. examined the effect of ACT containing cyclophosphamide, doxorubicin, and 5-fluorouracil (CAF) in a randomized phase III trial on 1477 postmenopausal women with hormone-receptor-positive, node-positive BC on disease-free survival (DFS) [20]. DFS was the primary, OS and toxicity were defined as secondary end points. The group receiving CAF and tamoxifen had better DFS compared to the group receiving tamoxifen alone (HR 0.76 (95% CI 0.64, 0.91), *P* = 0.002). Ten-year DFS for CAF-T and tamoxifen alone were 60% and 48%. The main weakness of this study was that only CHT regimen CAF was used and only 190 patients (13%) were older than 70 years. The unadjusted HRs for DFS in these subgroups suggest that the efficacy of CHT varies in dependence of nodal status and age. Patients with more than four positive nodes had greater benefit than patients with one to three positive nodes. Patients younger than 65 years might have had a greater degree of benefit than older patients (test for interaction *P* = 0.13, adjusted for prognostic factors). However, this was only shown in results of subgroup analysis unadjusted for other covariates. No comorbidities or geriatric assessments were taken into account.

Citron et al. explored ACT in 2.005 patients with node-positive BC to compare sequential doxorubicin, paclitaxel, and cyclophosphamide with concurrent doxorubicin and cyclophosphamide followed by paclitaxel for DFS and OS to determine whether the dose density of the agents improves DFS and OS and to compare toxicities [21]. Dose-dense treatment improved DFS (RR 0.74; *P* 0.010) and OS (RR 0.69; *P* 0.013). Four-year DFS was 82% for dose-dense regimens and 75% for the others. There was no difference in either DFS or OS between the concurrent and sequential schedules. There was no interaction between density and sequence. Dose density improved clinical outcomes significantly, and sequential CHT is as effective as concurrent CHT. The main weakness of this study was the small cohort of elderly with 298 patients being 60–69 years old and only 50 patients older than 70 years (3%). Although there seemed to be a benefit of dose density in all analyzed groups regardless of age and menopausal status, no stratified analysis was performed for age.

The multicenter, randomized phase III study of Perrone et al. investigated whether weekly docetaxel is more effective than standard CHT (CMF) in patients aged 65–79 years [22]. A geriatric assessment was carried out and the quality of life (QoL) was assessed. Two hundred ninety-nine patients were randomized. They received CMF (*n* = 152) or docetaxel (*n* = 147). After 70-month median follow-up, 109 DFS events were observed. Unadjusted hazard ratio (HR) of DFS for docetaxel vs. CMF was 1.21 [95% confidence interval (CI) 0.83–1.76, *P* = 0.32]; DFS estimate at 5 years was 0.69 with CMF and 0.65 with docetaxel. HR of death was 1.34 (95% CI 0.80–2.22, *P* = 0.26). There was no interaction between treatment arms and geriatric scales measuring patients’ ability or comorbidities. One death was attributed to CMF and two to docetaxel. Increasing age, impairment in instrumental daily living activities, number of comorbidities and docetaxel treatment were independently associated with severe non-hematological toxicity. QoL was worse with docetaxel for nausea–vomiting, appetite loss, diarrhea, body image, future perspective, side effects treatment, and hair loss items. In conclusion, a weekly dosage of docetaxel is not more effective than standard CMF as adjuvant treatment of older women with BC and worsens QoL and toxicity.

Crivellari et al. analyzed data from the International Breast Cancer Study Group Trial VII [23]. Postmenopausal patients with early, node-positive BC were randomized to receive either tamoxifen alone (306 patients) or tamoxifen plus three consecutive cycles of classical CMF (302 patients). Among the 299 patients who received at least one dose of CMF, women aged 65 years or older (*n* = 76) had higher grades of toxicity compared with women younger than 65 years (*n* = 223) (*P* = 0.004). Older patients also received less than the planed CMF dose compared with younger postmenopausal women (*P* = 0.0008). The subjective burdens of treatment were similar for younger and older patients based on quality-of-life measures (performance status, coping, physical well-being, mood and appetite). For older patients, the 5-year DFS rates were 63% for CMF plus tamoxifen and 61% for tamoxifen alone (hazards ratio [HR], 1.00; 95% confidence interval [CI], 0.65 to 1.52; *P* = 0.99). For younger patients, the corresponding 5-year DFS rates were 61% and 53% (HR, 0.70; 95% CI, 0.53 to 0.91; *P* = 0.008), but the test for heterogeneity of CMF effect according to age group was not statistically significant. The reduced effectiveness of CMF among older women could not be attributed to dose reductions according to dose received. In conclusion, the tolerability and effectiveness of CMF were both reduced for older patients compared with younger ones who received tamoxifen for 5 years.

The ICE II group explored different CHT regimens in patients older than 65 years with BC, who were randomized to receive four cycles of adjuvant epirubicin and cyclophosphamide (EC) or six cycles of CMF vs. six cycles of nab-paclitaxel and capecitabine (nPX) [24]. Primary endpoints were treatment discontinuations and overall frequency of adverse events. Thirteen of one hundred ninety-eight patients (6.6%) discontinued EC/CMF and sixty-nine of one hundred ninety-three patients (35.8%) discontinued nPX (*P* < 0.001) with one and five deaths observed during treatment. Grade 3 to 5 adverse events were more frequent among patients treated with EC/CMF (90.9%) than among those treated with nPX (64.8%) (*P* < 0.001), with hematological toxicities being more frequent with EC/CMF (88.4% vs. 22.3%; *P* < 0.001), but non-hematological toxicities (hand-foot syndrome, diarrhea, mucositis, fatigue, sensory neuropathy, thromboembolisms, and metabolic disorders) being more frequent with nPX (58.5% vs. 18.7%; *P* < 0.001). None of the geriatric scores (Charlson comorbidity index, Vulnerable Elders Survey [VES-13], Instrumental Activities of Daily Living [IADL], and G8) independently predicted grade 3 to 5 toxic events or treatment discontinuations. No differences in survival between the treatment groups were observed after 22.8 months. Compared with EC/CMF, treatment with nPX led to more treatment discontinuations and non-hematological toxicities in elderly patients with moderate or high-risk breast cancer. This trial stopped after phase 2 and did not proceed to phase 3 to adequately assess survival in both treatment arms. Thus, a statement about effectivity of the regimen is not possible. In conclusion, non-frail elderly patients with moderate or high-risk BC could be treated with taxane-based polychemotherapy. However, toxicities lead to lowered dosage and this sums up with higher risk of death during treatment, lowering potential survival benefits among elderly.

A meta-analysis performed by the Early Breast Cancer Trialists’ Collaborative Group (EBCTCG) that compared different polyCHT regimens for early BC analyzed the long-term outcome in 100.000 women from 123 randomized trials and found that patients benefit from the addition of taxane to anthracycline (AC)-based regimens in terms of risk of recurrence and BC mortality (RR 0.86, SE 0.04, two-sided significance (*P* = 0.0005) [25]. Both taxane and AC based, as well as higher cumulative-dosage AC-based regimens reduced BC mortality about 33%. This effect was largely independent of age, stage, grading and ER status. Chemoendocrine therapy both in pre- and postmenopausal women produced a greater proportional reduction in BC mortality than endocrine therapy alone. However, the apparent lack of relevance of age and ER status to proportional risk reduction is partly influenced by the type of regimen used. For instance, nearly half the evidence for older women with ER-positive disease came from a single trial (SWOG881411) of CAF in 1500 postmenopausal women with tamoxifen-treated ER-positive disease. This trial demonstrated that CHT significantly lowers BC mortality in this key patient group, adding benefits to effective endocrine therapy.

The absolute benefits at 10 or 15 years appear to be about three times as great for younger than for older women, and to be somewhat greater for recurrence than for mortality.

Another meta-analysis of the EBCTCG-group compared NACT with the same ACT among 4756 women with early BC in 10 different randomized trials between 1983 and 2002 [26]. They found that more than two thirds (1349 [69%] of 1947) of the patients with NACT had a complete or partial clinical response. The risk of local recurrence in 15 years was 21.4% for NACT vs. 15.9% for ACT, whereas no significant difference between NACT and ACT was found for distant recurrence (15-year risk 38.2% for NACT vs. 38.0% for ACT), as well as for BC mortality (34.4% vs. 33.7%; 1.06 [0.95–1.18]; *P* = 0.31) or death from any cause (40.9% vs. 41.2%; 1.04 [0.94–1.15]; *P* = 0.45). Age, nodal status, and planned local therapy did not affect the effect of CHT. Most of the CHT was AC-based (3838 [81%] of 4756 women). Therefore, it is not possible to evaluate the effects of other regimens.

The third meta-analysis performed by EBCTCG explored the effects of ACT and endocrine therapy for early BC on recurrence and 15-year survival rates analysis including 194 randomized trials [27]. Older regimens such as CMF, FAC or FEC were analyzed, whereas taxanes, trastuzumab, or modern aromatase inhibitors were not used. They found that 6 months of AC-based polyCHT reduces the annual BC death rate by about 38% in women younger than 50 years and by about 20% in older women aged 50–69 years, irrespective of the use of tamoxifen, the ER status, nodal status, or other tumor characteristics. Such regimens were significantly more effective than CHT with CMF. However, only few women older than 70 years were included in these trials (4.3% of the total cohort) and an even smaller number was treated with AC (1.42%). Due to the small number of elderly patients, the results are not reliably informative regarding a survival benefit in the subgroup of elderly patients.

Based on the EBCTCG trials, age and the benefit of ACT on relapse risk and mortality reduction were inversely associated, but it must be taken into account that the group of “elderly” represented less than 4% of the total postmenopausal population in that analysis. In the NSABP B-07 study, patients were divided by age with a cut-off of 50 years of age. Menopausal status was not considered and only few patients were older than 60 years. The Southwest Oncology Group (SWOG) trial investigated the regimens of melphalan or the combination of cyclophosphamide, fluorouracil, methotrexate, vincristine, and prednisone (CMFVP)and the comparison of tamoxifen vs. CMFVP in two different trials and the International Breast Cancer Study Group investigated CMF, regimens which are currently not applied any more. Patients older than 70 years were clearly underrepresented in these trials, the proportion of elderly patients in trials was lower than the real prevalence. That is the reason why the main weakness of this study is the heterogeneous data on regimens and dosages of the studies reviewed and the different definitions of “elderly”.

The review of Balducci et al. on the role of age in the treatment of BC, a benefit of ACT in women with hormone-receptor-negative BC was described, especially in HER2-positive tumors and of those regimens containing AC [28]. The authors recommend assessing the benefits and risks of ACT individually according to tumor-related factors such as stage, but also patient-related factors such as life expectancy and comorbidities.

Ring et al. also refer to the EBCTCG when analyzing four studies regarding the effects of CHT in older patients [29]. The benefit for women older than 70 years receiving CHT was similar in comparison to younger postmenopausal women, but this result was again not significant as the cohorts of older patients in the trials were too small, as stated above.

Two trials (ACTION and CASA) were supposed to analyze the benefits of ACT in women older than 70 and 65 years, respectively, with hormone-receptor-negative and/or high-risk hormone-receptor-positive tumors. Unfortunately, both trials were closed early due to poor recruitment.

The Cancer and Leukemia Group B (CALGB) 49,907 trial hypothesized that ACT is beneficial for older women and compared two treatment arms: CMF or AC vs. capecitabine. Of the total cohort of 633 patients, 61% were older than 70 years. Patients randomized to capecitabine were 2.4 times more likely to experience a relapse-free survival event and 2.1 times more likely to die than those receiving standard CHT.

### Toxicity of CHT in elderly patients

There are several studies analyzing the effect of different CHT regimens that also explored different toxicity profiles in elderly patients. Muss et al. compared toxicity of ACT in older and younger patients with node-positive BC and analyzed relapse-free and OS in three randomized trials [30]. Data from three trials were included: the Cancer and Leukemia Group B (CALGB) 8541, a trial that compared cyclophosphamide, doxorubicin, and fluorouracil in three different dose schedules; CALGB 9344, a trial that compared cyclophosphamide and doxorubicin with or without paclitaxel and CALGB 9741, a trial that compared the application of cyclophosphamide, doxorubicin, and paclitaxel every 2 vs. every 3 weeks. High-grade toxicities were compared among age groups. 3% of patients were older than 70 years, 7% of patients (*n* = 458) were aged older than 65 years, 38% were 51–64 years and 55% were younger than 50 years. 24 deaths (0.4%) were related to treatment; 7 of them in elderly patients (older than 65 years). In multivariate analyses, elderly patients were significantly more likely to have grade 4 hematologic toxicity, discontinue treatment because of toxicity or to die of acute myeloid leukemia or myelodysplastic syndrome. There were no significant differences in non-hematologic toxicity. Whereas older patients showed higher rates of hematologic toxicity and treatment-related deaths than younger patients, there was no increase in non-hematologic toxicity.

Fargeot et al. identified 338 women older than 65 years with axillary lymph node involvement with WHO performance status > 2; normal hematologic, hepatic, and renal functions; and no cardiac dysfunction [31]. Patients were randomly assigned to receive tamoxifen for 3 years or epirubicin for six cycles plus tamoxifen for 3 years. Anthracycline-based CHT and endocrine therapy improved survival in this cohort of elderly patients relative to tamoxifen alone (DFS rates were 69.3% and 72.6% respectively, 6-year OS was 79.1% and 79.8% respectively). The acute toxicity was mild: grade 2 neutropenia in 5.9%, grade 2 anemia in 2.0%, grade 3 nausea or vomiting in 4.6%, and grade 3 alopecia in 7.2%. Five cases (in five patients) of decreased left ventricular ejection fraction occurred after CHT. One patient died as a result of dysrhythmia related to carcinomatous lymphangitis. No secondary leukemia occurred. This study highlights the importance of CHT in terms of DFS. Moreover, this regimen seems to be safe for hematologic, non-hematologic, and cardiac toxicities.

## Discussion

While the subpopulation of elderly patients is increasing worldwide and represents a significant part of cancer patients and survivors, it is clearly underrepresented in most trials [1, 28, 32–34]. According to Qiusheng et al., the percentage of patients receiving AC vs. observation increased significantly from 2010 to 2018 [9]. The benefit of cytotoxic CHT in elderly patients is still controversial due to limited evidence from RCTs. However, even when elderly patients are included in clinical trials, they often do not address end points concerning elderly patients, such as preservation of body function, cognition, and independence [35]. Data from studies conducted in postmenopausal women are often extrapolated to the elderly population. The American Society of Clinical Oncology (ASCO) developed recommendations to improve the evidence for treating elderly patients with cancer. They encourage examining the effects of medical treatments in older adults in clinical trials [7]. The main challenges for evaluating the effectiveness of CHT in elderly cancer patients are small cohorts of patients in most RCTs due to bias-associated poorer recruitment and heterogeneity of treatment regimens in different studies. Furthermore, the definition of “elderly” range between 55 and 80 years in different trials. In older studies, “elderly” was defined as patients aged 55–69 years, with only a small number of participants older than 69 years. Therefore, the potential benefits of CHT remain uncertain in this age group. Furthermore, older studies lacked quantitative immunohistochemistry data, which could have helped in predicting risk and chemosensitivity.

This review suggests that there is evidence for the benefit of ACT and NACT in high-risk BC. Although some studies are suggesting that older patients have less aggressive tumor biology, others found no difference between subgroups of different ages when comparing overall pCR and surgical downstaging rates post NACT [16]. In specific subgroups such as TNBC, there were higher pCR rates in younger patients. In addition, downstaging to BCS eligibility was similar across age groups after NACT [36, 37]. Even authors who claimed that elderly women had lower overall rates of pCR demonstrated that this effect was not discernible in specific subgroups such as in patients with HER2-positive tumors [38, 39]. The multivariable logistic regression analysis in this study showed correlation between young age, clinical stage T4, invasive ductal cancer and poor differentiated BC with higher pCR rates. The pCR rate was significantly lower in elderly patients with HR + /HER2 − and TNBC. However, it was not possible to differentiate between luminal A and luminal B tumors. Nevertheless, similar pCR rates in elderly patients with HER2 + BC show that there might be other reasons for differences in response rates than age. Although pCR is used as a primary end point in many trials, it lacks formal validation as a surrogate end point for DFS and OS in patients with BC after NACT [40].

The EBCTCG overviews include older studies finding that the gain of CHT decreased with age in contrast to tamoxifen. However, only few women older than 70 years entered the CHT trials (4.3%) [41]. The reduced efficacy of CHT with increasing age might be associated with shorter expected life span, reductions in delivered dose, and possible differences in tumor biology resulting in reduced chemosensitivity [42, 43]. Furthermore, older regimens were examined (e.g., CAF or CMF) which are not applied any more. Therefore, the reduced response to CHT in elderly patients has to be assessed with caution. In contrast, possible benefits of CHT may be reasonably extrapolated to elderly women with high-risk node-positive BC [6, 44]. In comparison to TNBC, women with hormone-receptor-positive tumors have a smaller benefit from ACT, as demonstrated in the overview conducted by the CALGB [45]. They found higher treatment-related mortality in elderly patients. This points out the importance of investigating the benefit of CHT on elderly women with high-risk, hormone-positive BC in comparison to other alternatives (e.g., CDK-4/6-inhibitors, endocrine therapies) that are currently employed. Crivellari et al. also explored the question on treatment burdens, impairing quality of life. They found that the burdens of treatment were similar for younger and older patients based on quality-of-life measures (performance status, coping, physical well-being, mood, and appetite) [23]. Four CALGB trials on ACT found treatment-related mortality to be related to age: 0.2% in patients younger than 50, 0.7% in patients between 51 and 64 and 1.5% in patients older than 65 years. Age was also a significant risk factor for congestive cardiac failure in patients receiving ACT [29]. Though older patients showed higher rates of hematologic toxicity and treatment-related deaths than younger patients, there was no increase in non-hematologic toxicity [29]. Notably, quality of life was not addressed in most studies. Autonomy, functional status, and patient preference is key in the advisement of elderly patients and there is an enormous need to address the end point of QoL and functional status during and after BC treatment in future trials.

A study on the effect of NACT on pCR in a real-world setting showed lower pCR rates among the elderly. When response rates were compared and analyzed by subtypes, this difference seemed to be significant only for women with triple-negative tumors. Older women had lower rates of pCR but had comparable outcomes after NACT compared to those of younger women. Some recent real-world-setting trials included only patients older than70 years with TNBC and found a significant improvement in 5-year BCSS and 5-year OS in patients who received ACT in comparison with patients who did not receive CHT even when adjusting for age and comorbidities [11]. Crozier et al. reinforced this consideration, demonstrating improved OS with CHT in patients older than 70 years with TNBC regardless of nodal status and comorbidity score [10].

Regarding specific regimens, the studies analyzed in this review addressed the question of effectiveness of different CHT regimens and agents like AC-T, CMF, CAF, CEF, epirubicin alone [31], the comparison of AC and CMF [6, 18], EC or CMF vs. T and capecitabine [24]. The majority of studies did not evaluate the applied regimens [7, 9–12, 15–17, 28, 44, 46–49]. This heterogeneity leads to more difficulties when comparing studies and the efficacy, especially in elderly patients. The role of anthracyclines was specifically addressed in a French multicenter trial [31] showing the 6-year DFS was only slightly improved by CHT when added to endocrine therapy and no significant impact of anthracyclines could be detected on OS. According to Balducci et al., a survival benefit of ACT in women older than 70 years is detected when the risk of relapse is more than 10% and in women older than 80 years when the risk of relapse is more than 20% [28]. The CALGB 49907-trial showed that there is a benefit of treating patients aged over 65 years with a good performance status with standard polyCHT compared with single agent capecitabine CHT. An unplanned subset analysis in this trial showed that the major benefit for standard CHT was observed in patients with hormone-receptor-negative tumors [29]. It is more difficult to assess whether there is a benefit in patients with node-negative BC or high ER expression. Besides life expectancy and tolerability of CHT, other tumor-related parameters should be considered (tumor size, HER2 status, grading) [36].

One of the major limitations of this review is the heterogeneity in the definition of “elderly” patients in different studies, with age limits varying between 55 and 80 years. In some trials, no differentiation in subgroups was made according to steroid hormone expression levels and HER2 status. Another main problem is that trials were performed in different periods, 7 of these after 2015 [8–11, 15, 17, 46] and 20 before 2015 [6, 7, 12–14, 16, 18–20, 25, 26, 28, 31, 44, 47–49], which leads to the application of different agents and different dosages.

In conclusion, the use of adjuvant CHT in elderly patients with BC should be individualized, taking into account the estimated absolute benefit, life expectancy, and treatment tolerance. Tools for decision making such as genomic tests are available now and can help predicting risks for relapse, which can be taken into account in future studies. Geriatric assessment as well as patient involvement with assessment of quality of life may help in treatment decision. This review demonstrates the urgent need to improve the evidence for the appropriate treatment of elderly patients with BC.

## Data Availability

No datasets were generated or analyzed during the current study.

## References

[1] Talarico L, Chen G, Pazdur R (2004) Enrollment of elderly patients in clinical trials for cancer drug registration: a 7-year experience by the US Food and Drug Administration. J Clin Oncol 22(22):4626–4631. 10.1200/JCO.2004.02.175. (**PMID: 15542812**) 15542812 10.1200/JCO.2004.02.175

[2] Arias E (2004) United states life tables, 2002. Natl Vital Stat Rep 53:1–39 15580947

[3] Fisher B, Redmond C, Fisher ER et al (1986) Systemic adjuvant therapy in treatment of primary operable breast cancer: national surgical adjuvant breast and bowel project experience. Natl Cancer Inst Monogr 1:35–43 3534589

[4] Parry C, Kent EE, Mariotto AB et al (2011) Cancer survivors: a booming population. Cancer Epidemiol Biomarkers Prev 20:1996–2005 21980007 10.1158/1055-9965.EPI-11-0729 PMC3422885

[5] Deutsche Krebsgesellschaft (2019) Kennzahlen und Matrix zu den Brustkrebszentren. https://www.krebsgesellschaft.de/zertdokumente.html. Accessed 17 Feb 2019

[6] de la Haba Rodríguez JR, Méndez Vidal MJ, Gómez España A, Serrano Blanch R, Tejera Vaquerizo A, Barneto Aranda I, Aranda AE (2003) Adjuvant treatment in elderly patients with breast cancer: critical review of clinical practice. Am J Clin Oncol 26(4):398–401. 10.1097/01.COC.0000026484.30773.89. (**PMID: 12902894**) 12902894 10.1097/01.COC.0000026484.30773.89

[7] Hurria A, Levit LA, Dale W, Mohile SG, Muss HB, Fehrenbacher L, Magnuson A, Lichtman SM, Bruinooge SS, Soto-Perez-de-Celis E, Tew WP, Postow MA, Cohen HJ; American Society of Clinical Oncology (2015) Improving the evidence base for treating older adults with cancer: American Society of Clinical Oncology statement. J Clin Oncol 33(32):3826–3833. 10.1200/JCO.2015.63.0319. Epub 2015 Jul 20. PMID: 26195697 10.1200/JCO.2015.63.0319 26195697

[8] Williams AD, Dang CT, Sevilimedu V, Morrow M, Barrio AV (2022) Neoadjuvant chemotherapy for breast cancer in the elderly: are we accomplishing our treatment goals? Ann Surg Oncol 29(13):8002–8011. 10.1245/s10434-022-12206-8. Epub 2022 Jul 24. PMID: 35871672; PMCID: PMC10162805 10.1245/s10434-022-12206-8 PMC10162805 35871672

[9] Guo Q, Lan T, Lu Y, Hu Z, Xu H, Wang X, Shao X, Fu X (2023) Effectiveness of adjuvant chemotherapy for elderly patients with triple-negative breast cancer. Biomol Biomed 23(3):502–509. 10.17305/bjbms.2022.8163. (**PMID: 36408954; PMCID: PMC10171436**) 36408954 10.17305/bjbms.2022.8163 PMC10171436

[10] Crozier JA, Pezzi TA, Hodge C, Janeva S, Lesnikoski BA, Samiian L, Devereaux A, Hammond W, Audisio RA, Pezzi CM (2020) Addition of chemotherapy to local therapy in women aged 70 years or older with triple-negative breast cancer: a propensity-matched analysis. Lancet Oncol 21(12):1611–1619. 10.1016/S1470-2045(20)30538-6. (**PMID: 33271091**) 33271091 10.1016/S1470-2045(20)30538-6

[11] Janeva S, Zhang C, Kovács A, Parris TZ, Crozier JA, Pezzi CM, Linderholm B, Audisio RA, Olofsson BR (2020) Adjuvant chemotherapy and survival in women aged 70 years and older with triple-negative breast cancer: a Swedish population-based propensity score-matched analysis. Lancet Healthy Longev 1(3):e117–e124. 10.1016/S2666-7568(20)30018-0. (**Epub 2020 Nov 30 PMID: 36094184**) 36094184 10.1016/S2666-7568(20)30018-0

[12] Elkin EB, Hurria A, Mitra N, Schrag D, Panageas KS (2006) Adjuvant chemotherapy and survival in older women with hormone receptor-negative breast cancer: assessing outcome in a population-based, observational cohort. J Clin Oncol 24(18):2757–2764. 10.1200/JCO.2005.03.6053. (**PMID: 16782916**) 16782916 10.1200/JCO.2005.03.6053

[13] Kaplan HG, Malmgren JA, Atwood MK (2017) Triple-negative breast cancer in the elderly: prognosis and treatment. Breast J 23(6):630–637. 10.1111/tbj.12813. (**Epub 2017 May 9 PMID: 28485826**) 28485826 10.1111/tbj.12813

[14] Liedtke C, Hess KR, Karn T, Rody A, Kiesel L, Hortobagyi GN, Pusztai L, Gonzalez-Angulo AM (2013) The prognostic impact of age in patients with triple-negative breast cancer. Breast Cancer Res Treat 138(2):591–599. 10.1007/s10549-013-2461-x. (**Epub 2013 Mar 5 PMID: 23460246**) 23460246 10.1007/s10549-013-2461-x

[15] Kozak MM, Xiang M, Pollom EL, Horst KC (2019) Adjuvant treatment and survival in older women with triple negative breast cancer: a surveillance, epidemiology, and end results analysis. Breast J 25(3):469–473. 10.1111/tbj.13251. (**Epub 2019 Mar 29 PMID: 30925635**) 30925635 10.1111/tbj.13251

[16] Syed BM, Green AR, Nolan CC, Morgan DA, Ellis IO, Cheung KL (2014) Biological characteristics and clinical outcome of triple negative primary breast cancer in older women—comparison with their younger counterparts. PLoS ONE 9(7):e100573. 10.1371/journal.pone.0100573. (**PMID: 24999743; PMCID: PMC4085072**) 24999743 10.1371/journal.pone.0100573 PMC4085072

[17] Inwald EC, Ortmann O, Koller M, Zeman F, Hofstädter F, Evert M, Brockhoff G, Klinkhammer-Schalke M (2017) Screening-relevant age threshold of 70 years and older is a stronger determinant for the choice of adjuvant treatment in breast cancer patients than tumor biology. Breast Cancer Res Treat 163(1):119–130. 10.1007/s10549-017-4151-6. Epub 2017 Feb 15. PMID: 28205042; PMCID: PMC5387012 10.1007/s10549-017-4151-6 PMC5387012 28205042

[18] Pagani O, Gelber S, Simoncini E, Castiglione-Gertsch M, Price KN, Gelber RD, Holmberg SB, Crivellari D, Collins J, Lindtner J, Thürlimann B, Fey MF, Murray E, Forbes JF, Coates AS, Goldhirsch A; International Breast Cancer Study Group (2009) Is adjuvant chemotherapy of benefit for postmenopausal women who receive endocrine treatment for highly endocrine-responsive, node-positive breast cancer? International Breast Cancer Study Group Trials VII and 12–93. Breast Cancer Res Treat 116(3):491–500. 10.1007/s10549-008-0225-9. Epub 2008 Oct 25. PMID: 18953651; PMCID: PMC3589110 10.1007/s10549-008-0225-9 PMC3589110 18953651

[19] Inal A, Akman T, Yaman S, Demir Ozturk S, Geredeli C, Bilici M, Inanc M, Harputoglu H, Demirci U, Balakan O, Yesil Cinkir H, Alici S, Uysal Sonmez O, Goksel G, Gokoz Dogu G, Umit Unal O, Tamozlu T, Buyukberber S, Melih Boruban C, Isikdogan A (2013) Endocrine therapy alone vs. chemotherapy plus endocrine therapies for the treatment of elderly patients with endocrine-responsive and node positive breast cancer: a retrospective analysis of a multicenter study (Anatolian Society of Medical Oncology). J BUON 18(1):64–69. PMID: 23613390 23613390

[20] Albain KS, Barlow WE, Ravdin PM, Farrar WB, Burton GV, Ketchel SJ, Cobau CD, Levine EG, Ingle JN, Pritchard KI, Lichter AS, Schneider DJ, Abeloff MD, Henderson IC, Muss HB, Green SJ, Lew D, Livingston RB, Martino S, Osborne CK; Breast Cancer Intergroup of North America (2009) Adjuvant chemotherapy and timing of tamoxifen in postmenopausal patients with endocrine-responsive, node-positive breast cancer: a phase 3, open-label, randomised controlled trial. Lancet 374(9707):2055–2063. 10.1016/S0140-6736(09)61523-3. Epub 2009 Dec 10. PMID: 20004966; PMCID: PMC3140679 10.1016/S0140-6736(09)61523-3 PMC3140679 20004966

[21] Citron ML, Berry DA, Cirrincione C, Hudis C, Winer EP, Gradishar WJ, Davidson NE, Martino S, Livingston R, Ingle JN, Perez EA, Carpenter J, Hurd D, Holland JF, Smith BL, Sartor CI, Leung EH, Abrams J, Schilsky RL, Muss HB, Norton L (2003) Randomized trial of dose-dense versus conventionally scheduled and sequential versus concurrent combination chemotherapy as postoperative adjuvant treatment of node-positive primary breast cancer: first report of Intergroup Trial C9741/Cancer and Leukemia Group B Trial 9741. J Clin Oncol 21(8):1431–1439. 10.1200/JCO.2003.09.081. Epub 2003 Feb 13. Erratum in: J Clin Oncol 21(11):2226. PMID: 12668651 10.1200/JCO.2003.09.081 12668651

[22] Perrone F, Nuzzo F, Di Rella F, Gravina A, Iodice G, Labonia V, Landi G, Pacilio C, Rossi E, De Laurentiis M, D’Aiuto M, Botti G, Forestieri V, Lauria R, De Placido S, Tinessa V, Daniele B, Gori S, Colantuoni G, Barni S, Riccardi F, De Maio E, Montanino A, Morabito A, Daniele G, Di Maio M, Piccirillo MC, Signoriello S, Gallo C, de Matteis A (2015) Weekly docetaxel versus CMF as adjuvant chemotherapy for older women with early breast cancer: final results of the randomized phase III ELDA trial. Ann Oncol 26(4):675–682. 10.1093/annonc/mdu564. (**Epub 2014 Dec 8 PMID: 25488686**) 25488686 10.1093/annonc/mdu564

[23] Crivellari D, Bonetti M, Castiglione-Gertsch M et al (2000) Burdens and benefits of adjuvant cyclophosphamide, methotrexate, and fluorouracil and tamoxifen for elderly patients with breast cancer: the international breast cancer study group trial VII. J Clin Oncol 18:1412–1422 10.1200/JCO.2000.18.7.1412 10735888

[24] von Minckwitz G, Conrad B, Reimer T, Decker T, Eidtmann H, Eiermann W, Hackmann J, Möbus V, Marmé F, Potenberg J, Stickeler E, Simon E, Thomssen C, Huober J, Denkert C, Alfer J, Jackisch C, Nekljudova V, Burchardi N, Loibl S; German Breast Group Investigators. A randomized phase 2 study comparing EC or CMF versus nab-paclitaxel plus capecitabine as adjuvant chemotherapy for nonfrail elderly patients with moderate to high-risk early breast cancer (ICE II-GBG 52). Cancer 121(20):3639–3648. 10.1002/cncr.29506. Epub 2015 Jun 25. PMID: 26111104 10.1002/cncr.29506 26111104

[25] Early Breast Cancer Trialists' Collaborative Group (EBCTCG); Peto R, Davies C, Godwin J, Gray R, Pan HC, Clarke M, Cutter D, Darby S, McGale P, Taylor C, Wang YC, Bergh J, Di Leo A, Albain K, Swain S, Piccart M, Pritchard K (2012) Comparisons between different polychemotherapy regimens for early breast cancer: meta-analyses of long-term outcome among 100,000 women in 123 randomised trials. Lancet 379(9814):432–444. 10.1016/S0140-6736(11)61625-5. Epub 2011 Dec 5. PMID: 22152853; PMCID: PMC3273723 10.1016/S0140-6736(11)61625-5 PMC3273723 22152853

[26] Early Breast Cancer Trialists' Collaborative Group (EBCTCG) (2018) Long-term outcomes for neoadjuvant versus adjuvant chemotherapy in early breast cancer: meta-analysis of individual patient data from ten randomised trials. Lancet Oncol 19(1):27–39. 10.1016/S1470-2045(17)30777-5. Epub 2017 Dec 11. PMID: 29242041; PMCID: PMC5757427 10.1016/S1470-2045(17)30777-5 PMC5757427 29242041

[27] Early Breast Cancer Trialists' Collaborative Group (EBCTCG) (2005) Effects of chemotherapy and hormonal therapy for early breast cancer on recurrence and 15-year survival: an overview of the randomised trials. Lancet 365(9472):1687–1717. 10.1016/S0140-6736(05)66544-0. PMID: 15894097 10.1016/S0140-6736(05)66544-0 15894097

[28] Balducci L, Extermann M, Carreca I (2001) Management of breast cancer in the older woman. Cancer Control 8(5):431–441. 10.1177/107327480100800507. PMID: 11579340 10.1177/107327480100800507 11579340

[29] Ring A, Reed M, Leonard R, Kunkler I, Muss H, Wildiers H, Fallowfield L, Jones A, Coleman R (2011) The treatment of early breast cancer in women over the age of 70. Br J Cancer 105(2):189–193. 10.1038/bjc.2011.234. Epub 2011 Jun 21. PMID: 21694726; PMCID: PMC3142812 10.1038/bjc.2011.234 PMC3142812 21694726

[30] Muss HB, Berry DA, Cirrincione C, Budman DR, Henderson IC, Citron ML, Norton L, Winer EP, Hudis CA; Cancer and Leukemia Group B Experience (2007) Toxicity of older and younger patients treated with adjuvant chemotherapy for node-positive breast cancer: the cancer and Leukemia Group B Experience. J Clin Oncol 25(24):3699–3704. 10.1200/JCO.2007.10.9710. PMID: 17704418 10.1200/JCO.2007.10.9710 17704418

[31] Fargeot P, Bonneterre J, Roché H, Lortholary A, Campone M, Van Praagh I, Monnier A, Namer M, Schraub S, Barats JC, Guastalla JP, Goudier MJ, Chapelle-Marcillac I (2004) Disease-free survival advantage of weekly epirubicin plus tamoxifen versus tamoxifen alone as adjuvant treatment of operable, node-positive, elderly breast cancer patients: 6-year follow-up results of the French adjuvant study group 08 trial. J Clin Oncol 22(23):4622–4630. 10.1200/JCO.2004.02.145. Epub 2004 Oct 25. Erratum in: J Clin Oncol 23(1):248. PMID: 15505276 10.1200/JCO.2004.02.145 15505276

[32] Jemal A, Tiwari RC, Murray T et al (2004) Cancer statistics, 2004. CA Cancer J Clin 54:8–29 14974761 10.3322/canjclin.54.1.8

[33] Muss HB, Woolf S, Berry D et al (2005) Adjuvant chemotherapy in older and younger women with lymph node-positive breast cancer. JAMA 293:1073–1081 15741529 10.1001/jama.293.9.1073

[34] Balducci L, Beghe C (1999) Pharmacology of chemotherapy in the older cancer patient. Cancer Control 6:466–470 10758578

[35] Hurria A, Dale W, Mooney M, Rowland JH, Ballman KV, Cohen HJ, Muss HB, Schilsky RL, Ferrell B, Extermann M, Schmader KE, Mohile SG; Cancer and Aging Research Group (2014) Designing therapeutic clinical trials for older and frail adults with cancer: U13 conference recommendations. J Clin Oncol 32(24):2587–2594. 10.1200/JCO.2013.55.0418. PMID: 25071116; PMCID: PMC4129504 10.1200/JCO.2013.55.0418 PMC4129504 25071116

[36] Verdial FC, Mamtani A, Pawloski KR, Sevilimedu V, D'Alfonso TM, Zhang H, Gemignani ML, Barrio AV, Morrow M, Tadros AB (2022) The effect of age on outcomes after neoadjuvant chemotherapy for breast cancer. Ann Surg Oncol 29(6):3810–3819. 10.1245/s10434-022-11367-w. Epub 2022 Mar 5. PMID: 35246810; PMCID: PMC10901180 10.1245/s10434-022-11367-w PMC10901180 35246810

[37] Müller C, Schmidt G, Juhasz-Böss I, Jung L, Huwer S, Solomayer EF, Juhasz-Böss S (2021) Influences on pathologic complete response in breast cancer patients after neoadjuvant chemotherapy. Arch Gynecol Obstet 304(4):1065–1071. 10.1007/s00404-021-06018-6. Epub 2021 Mar 10. PMID: 33689016; PMCID: PMC8429372 10.1007/s00404-021-06018-6 PMC8429372 33689016

[38] von Waldenfels G, Loibl S, Furlanetto J, Machleidt A, Lederer B, Denkert C, Hanusch C, Kümmel S, von Minckwitz G, Schneeweiss A, Untch M, Rhiem K, Fasching PA, Blohmer JU (2018) Outcome after neoadjuvant chemotherapy in elderly breast cancer patients—a pooled analysis of individual patient data from eight prospectively randomized controlled trials. Oncotarget 9(20):15168–15179. 10.18632/oncotarget.24586. (**PMID: 29632634; PMCID: PMC5880594**) 29632634 10.18632/oncotarget.24586 PMC5880594

[39] Loibl S, Jackisch C, Lederer B, Untch M, Paepke S, Kümmel S, Schneeweiss A, Huober J, Hilfrich J, Hanusch C, Gerber B, Eidtmann H, Denkert C, Costa SD, Blohmer JU, Nekljudova V, Mehta K, von Minckwitz G (2015) Outcome after neoadjuvant chemotherapy in young breast cancer patients: a pooled analysis of individual patient data from eight prospectively randomized controlled trials. Breast Cancer Res Treat 152(2):377–387. 10.1007/s10549-015-3479-z. (**Epub 2015 Jun 25 PMID: 26109347**) 26109347 10.1007/s10549-015-3479-z

[40] Berruti A, Amoroso V, Gallo F, Bertaglia V, Simoncini E, Pedersini R, Ferrari L, Bottini A, Bruzzi P, Sormani MP (2014) Pathologic complete response as a potential surrogate for the clinical outcome in patients with breast cancer after neoadjuvant therapy: a meta-regression of 29 randomized prospective studies. J Clin Oncol 32(34):3883–3891. 10.1200/JCO.2014.55.2836. (**Epub 2014 Oct 27 PMID: 25349292**) 25349292 10.1200/JCO.2014.55.2836

[41] Abe O, Abe R, Enomoto K et al (2005) Effects of chemotherapy and hormonal therapy for early breast cancer on recurrence and 15-year survival: an overview of the randomised trials. Lancet 65:1687–1717 10.1016/S0140-6736(05)66544-0 15894097

[42] Hutchins LF, Unger JM, Crowley JJ et al (1999) Underrepresentation of patients 65 years of age or older in cancer-treatment trials. N Engl J Med 341:2061–2067 10615079 10.1056/NEJM199912303412706

[43] Kemeny MM, Peterson BL, Kornblith AB et al (2003) Barriers to clinical trial participation by older women with breast cancer. J Clin Oncol 21:2268–2275 12805325 10.1200/JCO.2003.09.124

[44] Pierga JY, Girre V, Laurence V, Asselain B, Diéras V, Jouve M, Beuzeboc P, Fourquet A, Nos C, Sigal-Zafrani B, Pouillart P; Institut Curie Breast Cancer Study Group (2004) Characteristics and outcome of 1755 operable breast cancers in women over 70 years of age. Breast 13(5):369–375. 10.1016/j.breast.2004.04.012. PMID: 15454191 10.1016/j.breast.2004.04.012 15454191

[45] Gennari R, Curigliano G, Rotmensz N et al (2004) Breast carcinoma in elderly women: features of disease presentation, choice of local and systemic treatments compared with younger postmenopasual patients. Cancer 101:1302–1310 15316944 10.1002/cncr.20535

[46] Plichta JK, Thomas SM, Vernon R, Fayanju OM, Rosenberger LH, Hyslop T, Hwang ES, Greenup RA (2020) Breast cancer tumor histopathology, stage at presentation, and treatment in the extremes of age. Breast Cancer Res Treat 180(1):227–235. 10.1007/s10549-020-05542-4. Epub 2020 Jan 24. PMID: 31980967; PMCID: PMC7066434 10.1007/s10549-020-05542-4 PMC7066434 31980967

[47] Giordano SH, Hortobagyi GN, Kau SW, Theriault RL, Bondy ML (2005) Breast cancer treatment guidelines in older women. J Clin Oncol 23(4):783–791. 10.1200/JCO.2005.04.175. (**PMID: 15681522**) 15681522 10.1200/JCO.2005.04.175

[48] Terman E, Sheade J, Zhao F, Howard FM, Jaskowiak N, Tseng J, Chen N, Hahn O, Fleming G, Huo D, Nanda R (2023) The impact of race and age on response to NAC and long-term outcomes in Black and White women with early-stage breast cancer. Breast Cancer Res Treat 200(1):75–83. 10.1007/s10549-023-06943-x. Epub 2023 Apr 29. PMID: 37120458; PMCID: PMC10224832 10.1007/s10549-023-06943-x PMC10224832 37120458

[49] Woodard S, Nadella PC, Kotur L, Wilson J, Burak WE, Shapiro CL (2003) Older women with breast carcinoma are less likely to receive adjuvant chemotherapy: evidence of possible age bias? Cancer 98(6):1141–1149. 10.1002/cncr.11640. (**PMID: 12973837**) 12973837 10.1002/cncr.11640

[50] Hurria A, Leung D, Trainor K, Borgen P, Norton L, Hudis C (2003) Factors influencing treatment patterns of breast cancer patients age 75 and older. Crit Rev Oncol Hematol 46(2):121–126. 10.1016/s1040-8428(02)00133-6. (**PMID: 12711357**) 12711357 10.1016/s1040-8428(02)00133-6

[51] Berry DA, Cirrincione C, Henderson IC et al (2004) Effects of improvements in chemotherapy on disease-free and overall survival of estrogen-receptor negative, node-positive breast cancer: 20-year experience of the CALGB US Breast Intergroup [abstract]. In: San Antonio breast cancer conference 2004

[52] Cabrera-Galeana P, Soto-Perez-de-Celis E, Reynoso-Noveron N, Villarreal-Garza C, Lara-Medina F, Alvarado-Miranda A, Espinosa-Fernandez JR, Esparza-Arias N, Mohar A, Bargallo-Rocha JE (2020) Real-world outcomes among older Mexican women with breast cancer treated with neoadjuvant chemotherapy. Oncologist 25(12):1023–1031. 10.1634/theoncologist.2019-0891. Epub 2020 Apr 28. PMID: 32275801; PMCID: PMC7938403 10.1634/theoncologist.2019-0891 PMC7938403 32275801

